# Supplementary material for: Slow-Paced Breathing Intervention in Healthcare Workers Affected by Long COVID: Effects on Systemic and Dysfunctional Breathing Symptoms, Manual Dexterity and HRV
Source: Biomedicines. 2024 Oct 3;12(10):2254. doi: 10.3390/biomedicines12102254 (PMC11505241; doi:10.3390/biomedicines12102254)
Supplement: Supplementary file 1 [file biomedicines-12-02254-s001.zip › Supplemental Table 4 NQ FU.docx]

| **Items** | **Controls**  **(n=53)** | **Long COVID T0 (n=58)** | **Long COVID T1 (n=33)** | **p-value** |
| --- | --- | --- | --- | --- |
| Chest pain | 1.19 (0.56) | 1.86 (1.09) | 1.63 (0.93) | **<0.001¥**  **0.041ᵻ** |
| Feeling of tightness | 1.84 (1.24) | 2.74 (1.46) | 2.23 (1.10) | **0.005¥**  **0.020 ᵻ** |
| Blurred vision | 1.23 (0.55) | 2.11 (1.13) | 1.87 (0.97) | **<0.001¥**  0.099 **ᵻ** |
| Vertigo | 1.26 (0.63) | 2.06 (1.35) | 1.97 (1.19) | **<0.001¥**  **0.041 ᵻ** |
| Feeling of being confused | 1.32 (0.73) | 2.17 (1.34) | 1.63 (0.93) | **<0.001¥**  **0.001 ᵻ** |
| Fast breathing alternating with deep, slow breathing | 1.05 (0.42) | 2.09 (1.20) | 2.00 (1.17) | **<0.001¥**  0.187 **ᵻ** |
| Shortness of breath | 1.21 (0.64) | 1.23 (1.00) | 1.10 (1.00) | **<0.001¥**  0.210 **ᵻ** |
| Chest constriction | 1.25 (0.71) | 1.94 (1.11) | 0.95 (1.00) | **<0.001¥**  0.123 **ᵻ** |
| Bloated feeling in the stomach | 2.05 (1.27) | 2.63 (1.40) | 1.14 (1.00) | **0.027¥**  0.358 **ᵻ** |
| Tingling sensation in the fingers | 1.65 (1.17) | 2.60 (1.48) | 2.43 (1.30) | **0.001¥**  0.148 **ᵻ** |
| Inability to take a deep breath | 1.26 (0.71) | 2.09 (1.31) | 1.93 (1.14) | **0.001¥**  0.500 **ᵻ** |
| Stiffness in the fingers or arm | 1.44 (0.94) | 2.17 (1.50) | 1.87 (1.36) | **0.028¥**  0.110 **ᵻ** |
| Tightness around mouth | 1 (0) | 1.20 (0.72) | 1.10 (0.55) | **0.039¥**  0.105 **ᵻ** |
| Cold hands or cold feet | 1.78 (1.16) | 2.89 (1.60) | 2.67 (1.35) | **0.007¥**  0.202 **ᵻ** |
| Heart palpitations | 1.63 (1.03) | 2.60 (1.19) | 2.50 (0.97) | **<0.001¥**  0.068 **ᵻ** |
| Feelings of anxiety | 1.84 (1.14) | 2.69 (1.41) | 2.40 (1.30) | **0.004¥**  0.132 **ᵻ** |
| **NQ final score** | **22.6 (7.89)** | **35.03 (12.23)** | **32.57 (8.64)** | **<0.001¥**  0.061 **ᵻ** |

**Table S4.** **Follow up results of the Nijmegen Questionnaire (NQ).** Data are reported as means (DS) since normally distributed and compared using ttest (¥ Long COVID vs Controls) and paired ttest (ᵻ Long COVID T0 vs Long COVID T1). In bold significant results ( p<0.05)
