# Supplementary material for: Slow-Paced Breathing Intervention in Healthcare Workers Affected by Long COVID: Effects on Systemic and Dysfunctional Breathing Symptoms, Manual Dexterity and HRV
Source: Biomedicines. 2024 Oct 3;12(10):2254. doi: 10.3390/biomedicines12102254 (PMC11505241; doi:10.3390/biomedicines12102254)
Supplement: Supplementary file 1 [file biomedicines-12-02254-s001.zip › Supplemental table 5 HRV_rev.docx]

| Item | Long COVID T0  (n=36) | Long COVID T1  (n=36) | P-value |
| --- | --- | --- | --- |
| mean HR (bpm) | 74.1 (63-80.5) | 73.3 (67.7 – 78.2) | 0.33 |
| RMSSD (msn) | 33 (23-55) | 33 (27 – 53) | 0.84 |
| SDNN (msn) | 57 (44-89) | 58 (44 – 88) | 0.94 |
| VLF power (< 0.04 Hz) | 6.7 (6-7.4) | 6.8 (6.1 – 7.5) | 0.28 |
| LF power (0.04 – 0.15 Hz) | 7.4 (7 - 8.3) | 7.7 (7 – 8.2) | 0.91 |
| HF power (0.15 – 0.40 Hz) | 6 (5.4-6.8) | 5.9 (5.2 – 6.8) | 0.31 |
| LF/HF | 1.23 (1.07 -1.9) | 1.31 (1.1 -1.9) | 0.7 |
| LF/VLF Right | 1.1 (1.0 -1.2) | 1.1 (1 – 1.2) | 0.13 |
| LF/VLF Left | 1.1 (1.0-1.2) | 1.1 (1 – 1) | 0.45 |
| THM power (ms²/Hz) | 258 (130-505) | 154 (49 -277) | 0.27 |

**Table S5**. **Heart rate variability (HRV) parameters in Long COVID subjects during slow paced breathing (SPB) at T0 and T1**. Data are reported as medians (IQR) since not normally distributed and analyzed using Wilcoxon sign-rank test. In bold significant results (p< 0.05). Comparison with control group are reported elsewhere [38],
