# Supplementary material for: Slow-Paced Breathing Intervention in Healthcare Workers Affected by Long COVID: Effects on Systemic and Dysfunctional Breathing Symptoms, Manual Dexterity and HRV
Source: Biomedicines. 2024 Oct 3;12(10):2254. doi: 10.3390/biomedicines12102254 (PMC11505241; doi:10.3390/biomedicines12102254)
Supplement: Supplementary file 1 [file biomedicines-12-02254-s001.zip › Supplemental tables 1 and 2 MUS Distress.docx]

**Table 1. Medically Unexplained Symptoms (M.U.S.) at T0 and T1.** Data are reported as Median (IQR) since not normally distributed and were analyzed using Wilcoxon Rank sum test (**¥** Long COVID vs Controls) and Wilcoxon Rank sign test (ᵻ Long COVID T0 vs T1). In bold significant results (p< 0.05).

|  | Controls (n= 53) | Long COVID T0 (n= 58) | Long COVID T1 (n=33) | p value |
| --- | --- | --- | --- | --- |
| Do you suffer from chronic and persistent fatigue? | 0 (0 -5) | 7 (4 - 8) | 7 (4 - 7) | **<0.001¥**  0.66 **ᵻ** |
| Have you been suffering from mood disorders for long? | 0 (0 - 0) | 4.5 (0 - 6) | 2 (0 - 7) | **<0.001¥**  0.68 **ᵻ** |
| Do you suffer from persistent insomnia or awakenings from sleep? | 0 (0 - 4) | 6 (0 - 8) | 5 (2 - 7) | **<0.001¥**  0.75 **ᵻ** |
| Do you suffer from persistent dowsiness during the day? | 0 (0 - 0) | 0 (0 - 7) | 3 (0 - 5) | **<0.001¥**  0.69 **ᵻ** |
| Have you been experiencing anxiety? | 0 (0 - 2) | 3 (0 - 7) | 2 (0 - 5) | **0.0012¥**  0.87 **ᵻ** |
| Have you been experiencing apathy? | 0 (0 - 0) | 0 (0 - 4) | 0 (0 - 1) | **0.007¥**  0.35 **ᵻ** |
| Have you been suffering from panic attacks? | 0 (0 - 0) | 0 (0 - 0) | 0 (0 - 0) | **0.012¥**  0.25 **ᵻ** |
| Have you been experiencing abnormal heart beats (arrhytmia or tachycardia) at rest? | 0 (0 - 0) | 3.5 (0 - 6) | 4 (0 – 6) | **<0.001¥**  0.87 **ᵻ** |
| Have you noticed changes in your appetite (excessive hunger or loss of appetite)? | 0 (0 - 0) | 0 (0 - 6) | 0 (0 – 6) | **<0.001¥**  0.61 **ᵻ** |
| Do you suffer from night hunger pangs (night binge eating disorder)? | 0 (0 - 0) | 0 (0 - 0) | 0 (0 – 0) | // |
| Have you been suffering from heartburn, stomach fullness, bloating or nausea? | 0 (0 - 4) | 2.5 (0 - 7) | 5 (0 – 6) | **0.007¥**  0.31 **ᵻ** |
| Do you suffer from irritable bowel syndrome? | 0 (0 - 0) | 0 (0 - 5) | 0 (0 – 0) | **<0.001¥**  0.4 **ᵻ** |
| Have you periodically been suffering from from constipation or altered bowel movement? | 0 (0 - 0) | 0 (0 – 5.5) | 0 (0 -0) | **<0.001¥**  0.78 **ᵻ** |
| Do you usually have cold hands or feet? | 0 (0 - 2) | 3.5 (0 - 7) | 4 (0 -7) | **0.003¥**  0.69 **ᵻ** |
| Do you suffer from altered perspiration during sleep? | 0 (0 - 0) | 1.5 (0 - 6) | 0 (0 – 7) | **0.004¥**  0.93 **ᵻ** |
| Do you often wake up in a bad mood? | 0 (0 - 0) | 0 (0 - 1) | 0 (0 - 5) | **0.002¥**  0.96 **ᵻ** |
| Have you been experiencing feelings of unjustified guilt? | 0 (0 - 0) | 0 (0 - 4) | 0 (0 – 3) | **0.013¥**  0.96 **ᵻ** |
| Do you have difficulties in experiencing pleasure or relief, as a result of positive events? | 0 (0 - 0) | 0 (0 – 4.5) | 0 (0 – 0) | **0.004¥**  0.25 **ᵻ** |
| Have you recently experienced significant weight loss? | 0 (0 - 0) | 0 (0 - 0) | 0 (0 – 0) | 0.094**¥**  0.75 |
| MUS score index | **48 (33 - 62)** | **55.5 (50.5-67)** | **58 (41 – 67)** | **<0.001¥**  0.39 **ᵻ** |
| n. MUS | **3 (1-5)** | **7.5 (5-11)** | **8 (6 – 11)** | **<0.001¥**  0.85 **ᵻ** |

**Table S2. Distress symptoms at T0 and T1.** Data are reported as Median (IQR) since not normally distributed and were analyzed using Wilcoxon Rank sum test (**¥** Long COVID vs Controls) and Wilcoxon Rank sign test (**ᵻ** Long COVID T0 vs T1). In bold significant results (p< 0.05).

|  | Distress symptoms | Controls  (n obs= 53) | Long COVID T0  (n obs=58) | Long COVID T1  (n obs=33) | P value |
| --- | --- | --- | --- | --- | --- |
| Cognitive symptoms | Have you been having problems with your memory for a long time? | 0 (0 - 0) | 7 (4 - 8) | 4 (0 – 7) | **<0.001¥**  0.06 ᵻ |
|  | Have you been having trouble concentrating for a long time? | 0 (0-0) | 6 (0 - 8) | 5 (0 – 7) | **<0.001¥**  0.84 **ᵻ** |
|  | Do you feel that you have a poor sense of judgement? | 0 (0 - 0) | 0 (0 - 0) | 0 (0 – 0) | **0.001¥**  0.13 **ᵻ** |
|  | Have you been seeing everything in a negative light for a long time? | 0 (0 - 0) | 0 (0 - 2) | 0 (0 – 5) | **<0.006¥**  0.66 **ᵻ** |
|  | Do you have recurring thoughts? | 0 (0 - 0) | 0 (0 - 7) | 0 (0 – 3) | 0.24¥  0.17 **ᵻ** |
|  | Have you been in a state of constant worry for a long period of time? | 0 (0 - 0) | 0 (0 - 7) | 0 (0 – 6) | **0.03¥**  0.34 **ᵻ** |
| Physical symptoms | Have you been suffering from persistent pain for a long period of time? | 0 (0 - 0) | 7 (0 - 8) | 4 (0 – 8) | **<0.001¥**  0.27 **ᵻ** |
|  | Have you been feeling nauseous or dizzy for a long time? | 0 (0 - 0) | 0 (0 - 5) | 0 (0 – 3) | **<0.001¥**  0.1 **ᵻ** |
|  | do you have frequent colds? | 0 (0 - 0) | 0 (0 - 0) | 0 (0 – 0) | 0.12¥  0.59 **ᵻ** |
| Emotional symptoms | Have you been experiencing feelings of irritability or anger for a long period of time? | 0 (0 - 0) | 0 (0 - 5) | 0 (0 – 5) | **0.006**  0.23 **ᵻ** |
|  | Have you been feeling agitated and/or unable to relax for a long time? | 0 (0 - 0) | 3 (0 - 6) | 3 (0 – 6) | **<0.001¥**  0.29 **ᵻ** |
|  | Have you been feeling overwhelmed for a long period of time? | 0 (0 - 0) | 0 (0 - 4) | 0 (0 – 5) | 0.35¥  0.63 **ᵻ** |
|  | Have you been feeling a sense of loneliness and/or isolation for a long period of time? | 0 (0 - 0) | 0 (0 - 0) | 0 (0 – 0) | 0.37¥  0.75 **ᵻ** |
|  | Have you been experiencing a state of melancholy and/or a general feeling of unhappiness for a long time? | 0 (0 - 0) | 0 (0 - 5) | 0 (0 – 4) | 0.06¥  0.66 **ᵻ** |
| Behavioral symptoms | Do you sleep too much or too little? | 0 (0 - 2) | 2 (0 - 7) | 3 (0 – 6) | 0.08¥  0.84 **ᵻ** |
|  | Do you have a tendency to isolate yourself from others? | 0 (0 - 0) | 0 (0 - 0) | 0 (0 – 0) | 0.77¥  0.75 **ᵻ** |
|  | Do you tend to procrastinate or neglect responsibilities? | 0 (0 - 0) | 0 (0 - 0) | 0 (0 – 0) | 0.77¥  1 **ᵻ** |
|  | Do you have a history of long term use of cigarettes, drinks or food for relaxation? | 0 (0 - 3) | 0 (0 - 5) | 0 (0 – 1) | 0.26¥  0.55 **ᵻ** |
|  | Have you been developing nervous habits (e.g. biting your nails) for a long time? | 0 (0 - 0) | 0 (0 - 0) | 0 (0 – 0) | 0.35¥  0.84 **ᵻ** |
|  | **distress score index** | **30 (0-48)** | **59.5 (46 – 70.5)** | **57 (35 – 66)** | **<0.001¥**  **0.05 ᵻ** |
|  | **n. distress** | **2 (0-4)** | **6 (4-9.5)** | **6 (4 – 8)** | **0.002¥**  0.19 **ᵻ** |
