# Supplementary material for: Slow-Paced Breathing Intervention in Healthcare Workers Affected by Long COVID: Effects on Systemic and Dysfunctional Breathing Symptoms, Manual Dexterity and HRV
Source: Biomedicines. 2024 Oct 3;12(10):2254. doi: 10.3390/biomedicines12102254 (PMC11505241; doi:10.3390/biomedicines12102254)
Supplement: Supplementary file 1 [file biomedicines-12-02254-s001.zip › Supplemental table 3_ISS_rev.docx]

|  | T0 (%)  N=58 | T1 (%)  N=33 | p value |
| --- | --- | --- | --- |
| asthenia | 56.3 | 31.3 | **0.007** |
| muscle weakness | 40.6 | 21.9 | **0.03** |
| recurrent fever | 9.4 | 6.2 | 1.0 |
| Diffuse pain | 18.7 | 15.6 | 1.0 |
| Arthromyalgia | 46.9 | 28.1 | **0.03** |
| Dyspnea | 37.5 | 31.2 | 0.50 |
| Persistent cough | 28.1 | 9.4 | **0.03** |
| Chest oppression | 28.1 | 21.9 | 0.50 |
| Tachycardia – palpitations | 37.5 | 28.1 | 0.25 |
| Blood pressure changes | 28.1 | 18.8 | 0.25 |
| Headache | 43.8 | 25 | **0.03** |
| Cognitive impairment | 59.4 | 34.4 | **0.007** |
| Peripheral neuropathy | 18.8 | 9.4 | 0.25 |
| ANS Alterations | 6.3 | 3.1 | 1.00 |
| Smell | 28.1 | 18.8 | 0.25 |
| Taste | 18.8 | 18.8 | 1.00 |
| Tinnitus | 15.6 | 9.4 | 0.5 |
| Earache | 12.5 | 12.5 | 1.00 |
| Dysphonia | 12.5 | 9.4 | 1.00 |
| Nausea | 15.6 | 0 | 1 |
| Abdominal pain | 5.6 | 6.3 | 0.25 |
| Diarrhea | 2.5 | 9.4 | 1.00 |
| Dyspepsia | 12.5 | 3.1 | 0.25 |
| gastroesophageal reflux | 6.3 | 3.1 | 1.00 |
| Abdominal disorders | 6.3 | 3.1 | 1.00 |
| Papule | 9.4 | 6.3 | 1.00 |
| Skin rash | 18.8 | 9.4 | 0.25 |
| Alopecia | 9.4 | 3.1 | 0.5 |
| Psoriasis | 3.1 | 3.1 | 1.0 |
| total no. of disorders | Mean (DS)  Median (IQR) | Mean (DS)  Median (IQR) |  |
| general symptoms | 1.97 (1.12)  1 (1-3) | 1.44 (1.13)  1 (1-2) | **0.016** |
| cardiovascular | 0.94 (0.91)  1 (0- 1.5) | 0.69 (0.9)  0 (0-1) | **0.030** |
| neurological | 1.28 (0.89)  1 (1-2) | 0.72 (0.85)  0.5 (0-1) | **< 0.001** |
| gastrointestinal | 0.72 (1.05)  0 (0-1) | 0.28 (0.52)  0 (0 – 0.5) | **0.016** |
| dermatological | 0.38 (0.55)  0 (0-1) | 0.22 (0.42)  0 (0-0) | 0.120 |

**Table S3. Long COVID symptoms as defined by Istituto Superiore di Sanità [32], assessed at T0 and after one month (T1).** Binary outcomes are reported as % and compared using McNemar test, continuous variables are reported as Mean (SD) and Median (IQR) and compared using t-test or Wilcoxon rank sum test if normally or not normally distributed, respectively.
